# Spatial effects should be allowed for in primary care and other community-based cluster RCTS

**DOI:** 10.1186/1745-6215-11-55

**Published:** 2010-05-14

**Authors:** Paul Silcocks, Denise Kendrick

**Affiliations:** 1 Clinical Trials Unit, University of Nottingham Medical School, Nottingham, UK; 2 Division of Primary Care, University of Nottingham Medical School, Nottingham, UK

## Abstract

**Background:**

Typical advice on the design and analysis of cluster randomized trials (C-RCTs) focuses on allowance for the clustering at the level of the unit of allocation. However often C-RCTs are also organised spatially as may occur in the fields of Public Health and Primary Care where populations may even overlap.

**Methods:**

We allowed for spatial effects on the error variance by a multiple membership model. These are a form of hierarchical model in which each lower level unit is a member of more than one higher level unit. Membership may be determined through adjacency or through Euclidean distance of centroids or in other ways such as the proportion of overlapping population. Such models may be estimated for Normal, binary and Poisson responses in Stata (v10 or above) as well as in WinBUGS or MLWin. We used this to analyse a dummy trial and two real, previously published cluster-allocated studies (one allocating general practices within one City and the other allocating general practices within one County) to investigate the extent to which ignoring spatial effects affected the estimate of treatment effect, using different methods for defining membership with Akaike's Information Criterion to determine the "best" model.

**Results:**

The best fitting model included both a fixed North-South gradient and a random cluster effect for the dummy RCT. For one of the real RCTs the best fitting model included both a random practice effect plus a multiple membership spatial term, while for the other RCT the best fitting model ignored the clustering but included a fixed North-South gradient. Alternative models which fitted only slightly less well all included spatial effects in one form or another, with some variation in parameter estimates (greater when less well fitting models were included).

**Conclusions:**

These particular results are only illustrative. However, we believe when designing C-RCTs in a primary care setting the possibility of spatial effects should be considered in relation to the intervention and response, as well as any explanatory effect of fixed covariates, together with any implications for sample size and methods for planned analyses.

## Background

Few examples of properly designed & analysed C-RCTs exist before 1978, but since then with developments in methodology and software such designs have become increasingly common [1].

It is well-recognised that a proper analysis of C-RCTs must allow for the clustered nature of the data to reduce the risk of a type 1 error. However other methodological biases may arise in such trials [2,3] and despite readily available advice [4] high quality design, analysis and reporting of C-RCTs remains limited [5-7].

One aspect which is frequently overlooked is the intrinsically spatial distribution of general practices and other community-based clusters, which have populations that are not only adjacent (and therefore likely to have shared exposures) but which actually overlap. In the context of GP Practices the overlap would be in the sense that a patient registered with one GP Practice may live next door to a patient registered with a completely different Practice (which is distinct from the kind of overlap that might occur if a single patient were registered with multiple Practices). The effect of proximity and possibly overlap and how to account for it has received little attention in the literature on C-RCT design and reporting [1-7].

In a time series, variations in incidence over time may be represented by a smooth "deterministic" trend combined with random noise which is likely to be autocorrelated - that is, observations which are closer together in time are more similar than observations further apart [8]. Spatial data may be represented similarly except that separation of observations occurs in two dimensions of space.

An additional complication familiar to geographers and others interested in modelling the spatial distribution of disease is that in spatial models the observations are typically not evenly distributed (unlike the regular periodic observations of many time series). One way to allow for spatial autocorrelation is through a spatial error model, one example of which is a multiple membership model.

**Figure 1:**
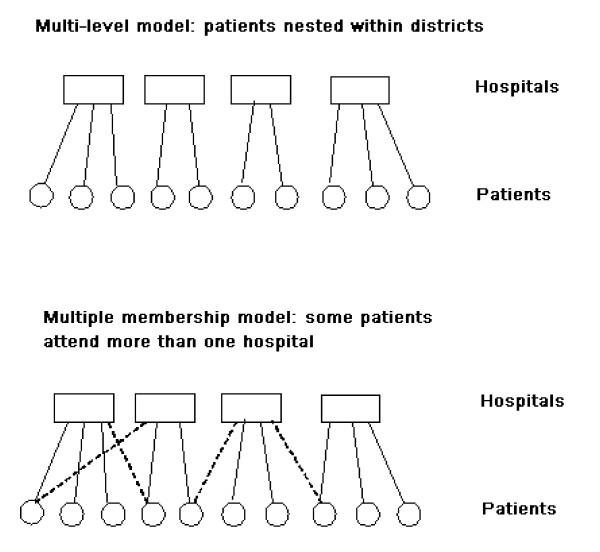
**Multi-level vs Multiple Membership models**.

A multiple membership model is therefore a form of hierarchical model in which each lower level unit can be a member of more than one higher level unit. Figure 1 illustrates the difference between a standard multilevel model (in which patients, for instance, each only attend one hospital within which they are "nested" in statistical terminology) and one form of a multiple-membership model in which some patients may attend more than one hospital.

The extent of such membership may be defined in different ways, depending on the data available and the purpose of the analysis. One way might be on the percentage of total in-patient stay; another might be on proximity. For spatial models in which the units of interest are geographically defined, membership may be defined in terms of adjacency or through Euclidean distance of centroids or in other ways such as the proportion of overlapping resident population. Such models may be estimated in WinBUGS or MLWin and also (for Normal, binary and Poisson responses) in Stata (version 10 or above).

## Methods

Three data sets were used: one dummy RCT and two real cluster-allocated studies for which one of us (DK) was chief investigator. The first of these allocated general practices within a single City and the other allocated general practices within a single County.

The dummy RCT was based on the Scottish male lip cancer data set [9]. This data set consists of 56 county districts in Scotland with observed and expected numbers of lip cancer cases over the period 1975-1980 together with a covariate indicating the extent of sun-exposure among the workforce. By courtesy of the Information Services Division of NHS National Services Scotland, these data were augmented with the x and y coordinates of each district's geographical centroid, enabling both a measure of distance to be calculated between each district and general east-west and north-south fixed effects to be included in each model.

The analysis of this data set by means of a Poisson multiple membership model presented by Browne [10] was first duplicated in Stata to confirm similarity of results.

We then randomly allocated each County district to one of two nominal treatments and the observed number of cases in those districts receiving one of the treatments was doubled to reflect the effect of a hypothetical intervention. It is important to remember that this not a real trial so that the actual nature of the outcome, the intervention and the covariate do not matter. The data are being used simply to provide realistic statistical data.

As implemented by Browne, the multiple membership function was defined as follows. For a given index district each adjacent district was assigned a membership weight of 1/n where n was the total number of such adjacent districts. The index district itself was not included thus enabling it to be represented by a separate random effect.

**Figure 2:**
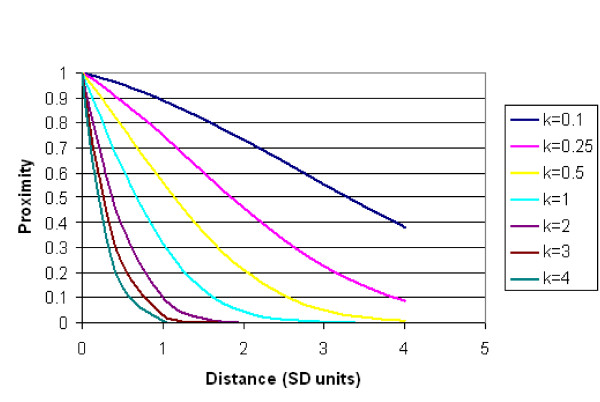
**Converting distance to proximity**. .

For our dummy RCT this procedure would not be feasible because some of the districts chosen might have no adjacent neighbours in the sample. However the influence of neighbouring Districts might not require actual adjacency. We therefore based membership on an ad hoc function of Euclidean distance between the District centroids. The function used to transform Euclidean distance to proximity was:

Where Distance is measured in Standard Normal Deviate units, Φ( ) denotes the standard Normal cumulative distribution function, and k is a parameter estimated by minimizing Akaike's Information Criterion (AIC) over a range of fitted models. Figure 2 illustrates the general effect on proximity due to different values of k. Proximity at a distance of zero was set to zero, to allow each District to have its own random effect, separate from that of the neighbours.

Figure 2 illustrates the flexible effect of different values of k on Proximity, ranging from a slow initial decay with a shoulder, through more rapid exponential decays tending towards the sharp cut-off similar to an adjacency-based proximity function which would take only values of 0 and 1.

For each index district in turn, membership of the other districts was defined as the proximity divided by the sum of all the proximities to the index. Again, the index district was omitted from the summation.

We then applied this function to the full Scottish lip cancer data set to identify firstly the value of k that gave the closest AIC to Browne's method, and secondly to identify the value that minimized the AIC.

While hypothetical in terms of the allocated "treatment", the resulting dummy C-RCT incorporates real covariate and proximity data as well as the response in the control arm (the response in the "intervention" arm is a multiple of what was actually observed). Such a C-RCT based on geographically defined clusters (as opposed to clusters such as hospitals or GP practices) might be performed in a Public Health context for road traffic measures or water fluoridation.

For each model (all using the same random number seed) the value of including a spatial gradient as a fixed effect was also assessed, and each model included the percentage of sun exposed workers as a covariate.

### The two actual trials

The first trial [11] evaluated the effect of a home safety intervention for children and included 18 practices in Nottingham randomly chosen for the intervention arm which were then pair-matched on Jarman score to control arm practices. Postcodes for place of residence that could be mapped to grid references were available for 1543 (73%) participants for whom outcome data were also available. The outcome variable was the number of injuries per individual modelled using Stata's xtmepoisson command. Unfortunately it was not possible to incorporate the matching in the analysis as the surviving data file from this old trial does not include the matching variable. However we do know that the original analysis ignored the matching as well. The original primary analysis in the paper was a logistic regression with outcome being occurrence of one or more injuries; however an additional Poisson regression was also presented with number of injuries as the response, which is the method adopted here as being more appropriate.

The second trial [12] evaluated an intervention to reduce baby walker use and included 44 practices (from a total of 46 randomised to intervention or control arms) in Nottinghamshire. Two practices did not recruit any participants and were therefore excluded from this analysis. Postcodes for place of residence that could be mapped to grid references were available for 982 (98%) participants for whom outcome data were also available. The outcome variable was binary (whether or not the family had a baby walker), modelled using the xtmelogit command.

The default analysis for the two trials was on an individual patient basis, with allowance for the clustering by means of a practice level random effect.

Because practice centroids derived from practice populations were not available, one of us (DK) converted participants postcodes into grid references using a file for East Midlands postcodes supplied by East Midlands Public Health Observatory. She then centred these values by subtracting the mean easting and northing respectively and then averaged these values over the patients within each practice to give a centroid for the practice based on participants (arguably perhaps more relevant), before passing these to PS for analysis. As with the Scottish data these centroids were used to estimate both distances between each practice and general east-west and north-south fixed effects.

All analyses were performed using Stata v10, and model selection was based on minimizing the AIC. The incomplete availability of postcode data was ignored for our purposes, because our interest lay in comparing estimated treatment effects with and without allowance for spatial effects rather than obtaining a "correct" reanalysis of the original trials. In addition we ignored the matching in the home safety intervention study partly because the original analysis did so and also because the matching variable could not be found in what remains of this now fairly old dataset.

For a given dataset and range of models, an AIC greater by four units or more than the minimum AIC is evidence of considerably less statistical support (as measured by the likelihood of the model), while differences in the range 0-2 indicate substantially the same support [13]. Because changes in the AIC may be regarded as changes in penalised log likelihoods, then in the same way as likelihood ratios do, they measure relative weight of evidence *given by the data in hand *(these are not significance-testing procedures). Benchmark values for likelihood ratios of 8 and 32 have been suggested [14] to distinguish between weak, moderate and strong relative evidence, hence a difference between two models in AIC of 4 points (corresponding to a (penalised) likelihood ratio of 54.6) is actually very strong evidence supporting one model over the other.

## Results

**Figure 3:**
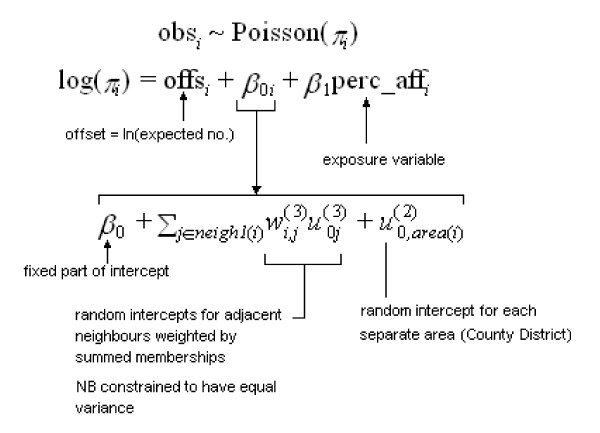
**Specification of Poisson Multiple Membership model**.

Figure 3 illustrates the model specification corresponding to Browne's analysis of the Scottish Lip cancer data. In this multiple membership model there is a fixed covariate - labeled perc_aff in the figure (standing for percent affected by sun-exposed occupations) - but there is no fixed spatial effect.

Initially we repeated Browne's analysis of the Scottish Lip Cancer data using Stata. The only variables for which there was any material difference were the the (common) random effect variance for the neighbours forming the multiple membership set, and the variance of the random intercept for districts themselves. MLWin gave values of 1.204 (0.493) and 0.056 (0.059) respectively, while corresponding Stata estimates were 0.980 (0.392) and 0.059 (0.055), values in brackets being the standard errors of each estimate. The fixed effect estimates and standard errors were similar even though Stata uses an approximate method to integrate out the random effects. In terms of choice of the parameter k we found that a value of 4.03 gave a model with AIC closest to the AIC of the adjacency membership model, albeit with rather different fixed parameter estimates (MLWiN Intercept = -0.308, Stata Intercept = -0.213; MLWin percent sunexposed = 0.049, Stata 0.041). For the dummy RCT we used a value of k = 1 which minimized the AIC at a value two units less than that for the adjacency multiple membership. This value of k gave a slope for percent sun-exposed very similar to Browne's although the intercept was 27% smaller in magnitude. We justify our choice by observing that the original adjacency membership function is not itself based on any a priori argument, nor on any formal model comparison and need not be considered necessarily "right".

Results for the dummy CRCT are displayed in Table 1. The best-fitting model included both a random area effect and a fixed North-South gradient. However the AIC for Treatment fixed effect + Multiple Membership random effect + fixed North-South gradient (denoted as Treat + MM + N/S grad), was only slightly greater, and in fact the four best-fitting models (all with an AIC no more than 4 units greater than the best model) all included a fixed North-South gradient. A Multiple Membership + random District model was rather worse with an AIC just over 4 units greater than the best fitting model, but still with a comparable estimate of the treatment effect. However other models that ignored any kind of random effect faired much less well: not only did they underestimate the magnitude of the treatment effect, these models included ones with both the smallest and the largest standard errors (a fivefold range in variance) while the four best-fitting models all had similar standard errors near the middle of this range.

**Table 1:** Results from a Dummy CRCT.

					AIC
Model	k	b(Treat)*	se	AIC	Difference
Treat + Random District + N/S	-	0.784	0.217	232.22	0

Treat + Random District + N/S + E/W	-	0.748	0.212	232.27	0.05

Treat + Random District + MM + N/S	1	0.786	0.212	234.02	1.8

Treat + Random District + MM + N/S + E/W	1	0.748	0.212	234.27	2.05

Treat + Random District + MM	1	0.753	0.203	236.34	4.12

Treat + Random District	-	0.491	0.262	247.86	15.64

Treat + N/S + E/W	-	0.592	0.116	285.20	52.98

Treat + N/S + E/W using robust se		0.592	0.233	285.20	52.98

Treat + N/S	-	0.613	0.117	287.65	55.43

Treat + N/S using robust se		0.613	0.246	287.65	55.43

Treat + robust se	-	0.247	0.260	361.18	128.96

True value		0.693	-	-	-

* expressed as log_e_(hazard ratio) in all models, including % sun exposed (perc_aff) as a covariate

Tables 2 and 3 display results for the two real trials. In Table 2 (home safety intervention study) the likelihood ratio test for the random effect being zero was highly significant and illustrated by the great difference in AIC between the standard Poisson model and the one including a random practice term. Nevertheless the minimum AIC model was one that included both a random Practice and a multiple membership term. Inclusion of fixed spatial gradients (east-west and north-south) did not produce a major effect on the AIC, but did diminish the estimated treatment effect by an order of magnitude. Not only this, but some models actually reversed the sign of the estimated treatment effect, and this was also seen in the model with a random practice effect and the Poisson model with robust (cluster) standard error. As with the dummy RCT the best fitting models had similar standard errors, but the remainder mostly had much greater standard errors for the treatment effect. The point estimate and confidence interval with the random Practice effect was 1.00 (CI 0.76	 to 1.32) similar to the values quoted in the paper (1.00; CI 0.78 to 1.28)

**Table 2:** Results from "Home safety intervention" trial.

				AIC
Model	b(Treat)	se	AIC	Difference
Rand Prac + MM	-0.0147	0.098	2724.86	0.00

Rand Prac + MM + N/S	-0.0098	0.098	2726.27	1.41

Rand Pract + MM + E/W + N/S	-0.0095	0.099	2728.27	3.41

Random pract^a^	0.0015	0.140	2735.66	10.80

Random practice + N/S	0.015	0.140	2737.18	12.33

Robust se (pract as cluster)	-0.0278	0.136	2761.04	36.18

Ignoring clustering^b^	-0.0278	0.076	2761.038	36.18

LR test for random effect, model (a) vs model (b): chisquared 2(01) = 27.38 P = 0.0000

**Table 3:** Results from "Baby Walker" trial.

				AIC
Model	b(Treat)	se	AIC	Difference
Ignore clustering N/S gradient only	-0.660	0.141	1245.57	0.00

Ignore clustering + N/S + E/W	-0.651	0.141	1247.09	1.53

Rand Pract + N/S	-0.655	0.148	1247.48	1.91

Rand Pract + N/S + E/W	-0.646	0.148	1249.03	3.46

Ignore clustering^a^	-0.618	0.138	1254.50	8.93

Robust se (pract as cluster)	-0.618	0.164	1254.50	8.93

Rand Pract^b^	-0.629	0.167	1255.29	9.73

Rand Pract + MM	-0.609	0.158	1256.92	11.35

LR test for random effect, model (a) vs model (b): chisquared 2(01) = 0.06 P = 0.4017

In the baby walker study (Table 3) by contrast, the likelihood ratio test for the random effect being zero was non-significant (P = 0.4). The clustering therefore appeared to have little effect in this case, and in fact the lowest AIC model had neither a random effect nor a multiple membership term, although a spatial effect in the form of a North-South gradient did greatly improve the fit over a model with no spatial component. On the other hand the extent to which the estimated treatment effect varied between the models was much less than in the home safety intervention study. Again, the best fitting models had similar, non-extreme, standard errors, and the model with Practice as random effect (unadjusted for other variables) gave an odds ratio and confidence interval (0.54; CI 0.39 to 0.74) similar to the original unadjusted analysis for "having a baby walker".

## Discussion

The results of this study can only of course be illustrative and will not necessarily apply to all C-RCTs, however, we believe that the possibility of spatial effects should be considered when designing any C-RCT in a primary care setting or planning its analysis.

For this study we have also assumed that the primary interest is in the treatment fixed effect and its standard error, for which the Laplacian method used by Stata is fast but may underestimate random effects which might cause a problem if treatment by centre interactions are to be studied. However if necessary this problem can be avoided by use of WinBUGS or MLWin.

On the other hand it is useful to be able to evaluate different forms of proximity weighting and random effects models rapidly within a familiar computing environment without the need to learn new, infrequently used, packages.

For our dummy RCT representing a C-RCT performed in a population of about 5 million, a random treatment effect combined with a fixed North-South gradient performed best and there was a moderate sensitivity to model choice of the estimated treatment effect. For the home safety intervention study the minimum AIC model was one that included both a random practice and a multiple membership term, and although addition of a fixed spatial gradient had relatively little effect on the AIC, the estimated treatment effect was very sensitive to model choice, even to the extent of reversing its sign.

By contrast in the baby walker study the estimated treatment effect was relatively insensitive to model choice and the best fitting model ignored random effects altogether, although it did include a fixed spatial term.

In all three studies both the point estimate and its standard error were affected by the model choice; while the best-fitting (minimum AIC) models did not have the smallest standard errors, these were not the largest either, and this benefit was apparent even if the range of point estimates was not large.

We are not sure how we would have allowed for the matching in the home safety trial in addition to the spatial effect, had it been available (there is no guarantee of course that matched practices would have been close together spatially). Because this was pair-matching, use of a fixed effect dummy variable to denote each matched pair would have been inadvisable, so we would probably have treated the matching variable as an additional random effect.

Other rules could be used for defining the membership function, but we have not explored them here. One topic of potential interest would be the extent to which other ways of defining spatial relationships in C-RCTs based in primary care influence conclusions, and in particular the effect of using individual participant grid references as opposed to those of Practices.

It is of some interest to speculate on the reasons why a North-South effect may have been important in the baby walker study but not in the home safety intervention study, although this is of necessity no more than a post-hoc rationalization. One possibility is that the baby walker study included a wider and possibly more diverse population, whereas the home safety intervention study included practices within a single city, where the north/south gradient in terms of deprivation would be less marked.

In addition, random effects models (of which a MM is one kind) can be used as a catch-all for various unmeasured effects and conceivably if these could be identified in advance they could be handled as fixed effects and make the random effect less relevant - and the fixed effect more specific, if for instance the North-South gradient could be replace by a more direct measure of deprivation. A further improvement could be obtained if individual-level covariates were included. Lastly both the kind of intervention and the nature of the response variable may influence whether allowance for spatial effects is likely to be important, and therefore allowed for in sample size estimation and in the statistical analysis plan.

## Conclusions

In brief we conclude that:

1. spatial effects may well need to be allowed for in the design and analysis of C-RCTs.

2. the optimum analysis method may involve either a multiple membership and or a fixed spatial covariate and the choice should be identified by a pre-specified process of model selection.

3. ignoring spatial effects may affect model fit, estimated parameter values and their standard errors.

## Competing interests

Paul Silcocks - None. Denise Kendrick - was Chief investigator of the two previously published Trials used in this paper.

## Authors' contributions

PS conceived the study, performed the analyses and drafted the manuscript. DK provided data on the studies used, generated the anonymous analysis data set used by PS and revised and jointly approved the final version of the manuscript.
